# World Health Organization Regional Office for Africa Weekly Bulletin on Outbreaks and Other Emergencies

**DOI:** 10.3201/eid2407.180573

**Published:** 2018-07

**Authors:** Benido Impouma, Brett N. Archer, Okot Charles Lukoya, Esther L. Hamblion, Ibrahima Socé Fall

**Affiliations:** World Health Organization Regional Office for Africa, Cité du Djoué, Democratic Republic of the Congo

**Keywords:** disease outbreaks, communicable diseases, emergencies, disasters, Africa, communication, WHO Weekly Bulletin on Outbreaks and Other Emergencies, World Health Organization, Regional Office for Africa

The basis for effective public health action is accurate and timely information (*1*). During emergencies, a fundamental public health tool is rapid, proactive, and transparent communication. Information gathered and disseminated during emergencies not only guides public health authorities but also encourages communities to adopt protective behaviors, triggers a heightened level of disease surveillance across borders, and reduces confusion among national authorities and communities (*2*). Although the International Health Regulations (2005) have provided a strong system for urgent communications (*3*), these alerts often remain siloed among a few parties. For several decades, lack of information sharing or incompatible communication systems have remained paralyzing factors for complex emergency responses (*4*). Indeed, among the lessons from the 2014–2016 Ebola virus disease epidemic was the value of effective communication and transparency in reporting (*1*).

The Transformation Agenda of the World Health Organization (WHO) Secretariat in the African Region 2015–2020 has 4 major objectives, 1 of which is improved strategic and effective communication (*5*). In keeping with this objective, in March 2017, the WHO Regional Office for Africa launched the Weekly Bulletin on Outbreaks and Other Emergencies (the Bulletin). The Bulletin (Figure) is not intended to compete with or replace the more traditional communication strategies, which range from targeted risk communications and media to peer-reviewed scientific publications. Rather, the Bulletin aims to bridge the gaps within this spectrum, providing real-time actionable updates on the status of new and ongoing events, while highlighting actions taken and gaps that need addressing by Member States and partners.

**Figure:**
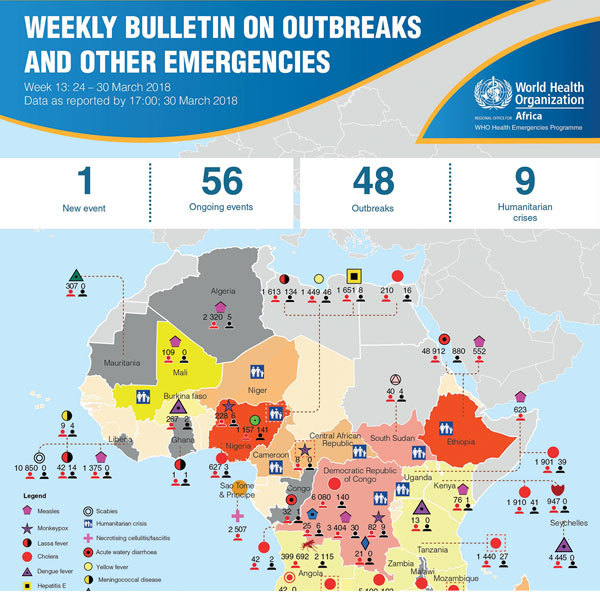
Cover of recent edition of the Weekly Bulletin on Outbreaks and Other Emergencies, published by the World Health Organization Regional Office for Africa.

However, providing regular communications on emergencies in the African region is no easy task. The sheer scope and magnitude of emergencies presents an enormous challenge. Capacity on the ground is often limited, and investigations and response efforts often take precedence over information dissemination. Communications must also proceed amid a great deal of uncertainty; events are often rapidly evolving or subjected to political sensitivities. For too long, these challenges have precluded timely communications about emergencies in the African region; information often remains unpublished, is published in retrospect, or is credited to authors outside the region.

The Bulletin provides a platform for overcoming these challenges. Through participation in the writing process, it provides a mechanism for WHO country offices to rapidly communicate updates to a wide audience. In 2017, the Bulletin published 43 editions, including 245 articles, and disseminated them directly to a growing readership of ≈2,000 members and posted them on social media and public health information websites, including but not limited to ProMED-mail, Outbreak News Today, and ACAPS (Assessment Capacities Project).

With the support of WHO Member States, it is our hope that the Bulletin will continue to play a major role in improving communication in the region. We welcome readers to join our mailing list.
